# Development of a pneumatically driven active cover lid for multi-well microplates for use in perfusion three-dimensional cell culture

**DOI:** 10.1038/srep18352

**Published:** 2015-12-16

**Authors:** Song-Bin Huang, Dean Chou, Yu-Han Chang, Ke-Cing Li, Tzu-Keng Chiu, Yiannis Ventikos, Min-Hsien Wu

**Affiliations:** 1 Graduate Institute of Biochemical and Biomedical Engineering, Chang Gung University, Taoyuan, Taiwan, R.O.C; 2 Institute of Biomedical Engineering, University of Oxford, Oxford, U.K; 3 Department of Engineering Science, University of Oxford, Oxford, U.K; 4 Department of Orthopaedic Surgery, Chang Gung Memorial Hospital, Linko, Taiwan, R.O.C; 5 Department of Chemical and Materials Engineering, Chang Gung University, Taoyuan, Taiwan, R.O.C; 6 Department of Mechanical Engineering, University College London, London, U.K

## Abstract

Before microfluidic-based cell culture models can be practically utilized for bioassays, there is a need for a transitional cell culture technique that can improve conventional cell culture models. To address this, a hybrid cell culture system integrating an active cover lid and a multi-well microplate was proposed to achieve perfusion 3-D cell culture. In this system, a microfluidic-based pneumatically-driven liquid transport mechanism was integrated into the active cover lid to realize 6-unit culture medium perfusion. Experimental results revealed that the flow of culture medium could be pneumatically driven in a flow-rate uniform manner. We used the system to successfully perform a perfusion 3-D cell culture of mesenchymal stem cells (MSCs) for up to 16 days. Moreover, we investigated the effects of various cell culture models on the physiology of MSCs. The physiological nature of MSCs can vary with respect to the cell culture model used. Using the perfusion 3-D cell culture format might affect the proliferation and osteogenic differentiation of MSCs. Overall, we have developed a cell culture system that can achieve multi-well microplate-based perfusion 3-D cell culture in an efficient, cost-effective, and user-friendly manner. These features could facilitate the widespread application of perfusion cell culture models for cell-based assays.

In life science research, *in vitro* cell-based assays have been widely utilized in drug screening^1^,^2^, toxin testing^3^,^4^, evaluation of the biocompatibility of materials^5^,^6^, and the study of cell biology^7^,^8^. Such cell-based assays can provide more biologically meaningful information than simplified biochemical assays. Cell-based assays also have the potential to be conducted in a more high-throughput and cost-effective manner than animal tests. Currently, the most commonly adopted cell culture model for biological assays is the static monolayer cell culture, in which the cells attach, spread, and grow on a 2-dimensional (2-D) surface and the culture medium is supplied manually at intervals during the period of cell culture (e.g., the use of multi-well microplates as cell culture vessels). The key advantages of such a conventional cell culture model are its lower cost and ease of operation in terms of preparation and observation. Nevertheless, this model has inherent shortcomings, including its inability to provide well-defined and biologically relevant culture conditions due to the static and 2-D monolayer cell culture format that is used^9^. These shortcomings could therefore prevent scientists from conducting precise and physiologically meaningful assays.

Microfluidics refers to the technology that allows scientists to manipulate tiny amounts of fluids using micro-scale structures with dimensions of the order of tens to hundreds of micrometers^10^. With the current rapid progress in microfluidic technology, microfluidic devices have been utilized as versatile tools for various cell culture-based assays, which have been extensively reviewed elsewhere^9^. For example, microfluidic-based cell culture devices have been successfully used in drug testing^11^,^12^, the study of biomaterials^13^,^14^, tissue engineering^15^,^16^, and the fundamental study of cellular physiology^17^,^18^. As a promising alternative to conventional cell culture methods, the use of microfluidic-based cell culture devices has several intrinsic advantages. Due to their miniaturized features, microfluidic cell culture systems consume fewer experimental resources than conventional culture systems, thus making high-throughput cell-based assays feasible. More importantly, due to their small dimensions, microfluidic cell culture systems offer immense promises for the provision of more well-defined^19^ and biomimetic culture conditions^20^ that can be used to develop more precise and physiologically relevant cell-based assays. Moreover, the liquid flow in a microfluidic system can be used to create a perfusion cell culture in which fresh and spent medium can be supplied and removed in a continuous manner. Such a perfusion cell culture format is generally believed to provide more stable and thus definable culture conditions for more precise bioassay work compared with conventional static cell cultures^19^.

Although microfluidic-based cell culture systems possess several advantageous features, the application of these emerging cell culture tools has not resulted in an evolutionary shift from the use of conventional cell-based assay methods^9^. Most of the demonstrations published academically in this area are only at the proof-of-concept stage, and many technical issues must still be adequately addressed before these systems can move from conceptual demonstration to actual application. First, the design of a microfluidic system for cell culture should enable biologists to conduct experiments without encountering numerous technical barriers. Secondly, when a novel cell culture methodology is adopted, the interpretation of the resulting data is challenging in terms of reconciling differences with data obtained from similar assays based on conventional cell culture techniques^21^. To address this issue, more fundamental research is required to bridge the gap between conventional and novel protocols. The third technical issue is related to the availability of detection methods that are capable of reading out the results of a cell culture-based assay. Ideally, detection should be performed in a simple, efficient, and high-throughput manner, as commercial microplate readers do for multi-well microplate-based cell culture practices. However, the current development in this area (e.g., the integration of bio-sensing components in microfluidic systems) is at its proof-of-concept stage, and the relevant detection mechanisms incorporated in microfluidic systems are, by and large, not sufficiently robust to immediately meet the requirements of practical applications.

Before we can practically utilize microfluidic cell culture systems to conduct more efficient, precise, and physiologically meaningful cell-based assays, there is an urgent need for a transitional cell culture model that can practically tackle the technical disadvantages that are present in conventional static 2-D monolayer cell culture models. To address this issue, this study proposes a hybrid system that integrates microfluidic technology with conventional multi-well microplate-based cell culture methods. Briefly, the integrated cell culture system consists of a 24-well microplate for accommodating 3-D cell culture samples and a pneumatically driven active cover lid that can seal the multiple wells in the microplate to form a closed system for cell culture. In the design, the active cover lid functions not only as a top lid to seal the multi-well microplate but, more importantly, to drive and control the culture medium to flow between the wells of the microplate to create a perfusion cell culture. The design of this device largely eliminates the need for costly and bulky liquid pumping equipment (e.g., bench-top syringe pumps) and for the labour-intensive works involved in setting up the tubing interconnections that are required in current perfusion cell culture practices. Due to its 3-D and perfusion cell culture formats, moreover, the cell culture model we describe can provide a more biomimetic^21^ and stable^19^ culture environment, thus enabling researchers to conduct more physiologically relevant and precise cell-based assays compared with the conventional 2-D static cell culture counterparts. With the aid of recent progress in laboratory equipment automation and high-throughput screening (HTS) techniques, furthermore, several types of laboratory equipment (e.g., microplate readers and liquid-handling machines) are compatible with multi-well microplate-based assays. Therefore, the proposed hybrid cell culture system could also make high-throughput detection and analytical tasks more efficient and less technically demanding. Overall, the proposed cell culture device allows biologists to carry out perfusion 3-D cell culture-based assays in an economical, user-friendly, and efficient manner.

In this work, we describe the design and fabrication of a pneumatically driven active cover lid that can be used to create a multi-well microplate-based perfusion 3-D cell culture. Additionally, the operating conditions for the use of this culture system were optimized, and its working performance was evaluated. As a whole, the computational simulation results revealed that the pneumatic pressures exerted were able to homogeneously distribute within the designed pneumatic microchannel within a very short time frame (1.0E-3 sec). This demonstrates the potential of this device to use pneumatic pressure to simultaneously and uniformly drive 6 culture medium flows. This was further experimentally verified, and it was shown that the culture medium flows can be pneumatically driven in a flow-rate uniform manner [coefficient of variation (CV): 1.1%]. In addition to development of the device, investigations were carried out to fundamentally explore the effect of different cell culture models on the outcomes of cell culture-based assays and to thereby bridge the gap between conventional and novel protocols. As a demonstration case, the influence of cell culture models [(i) a static 2-D cell culture; (ii) a static 3-D cell culture; (iii) a perfusion 2-D cell culture; and (iv) a perfusion 3-D cell culture using the proposed active cover lid] on cell viability and proliferation as well as on the osteogenic differentiation of mesenchymal stem cells (MSCs) was investigated. The results showed that the physiological nature of MSCs varies depending on the cell culture model used. Within the experimental conditions explored, the use of a perfusion or 3-D cell culture format appears to upregulate the proliferation and osteogenic differentiation of MSCs. Overall, we have developed an efficient, cost-effective, and user-friendly cell culture system for multi-well microplate-based perfusion 3-D cell culture. These features hold great promise for the widespread application of perfusion cell culture models in cell-based assays.

## Results and Discussion

### Characteristics of the active cover lid for multi-well microplate-based perfusion 3-D cell culture

The prevalent challenges facing the use of conventional static 2-D monolayer cell culture and of advanced cell culture methods (e.g., microfluidic-based cell culture models) were described earlier in this work. Before we can address the practical application issues concerning emerging microfluidic cell culture models, there is an urgent need for a transitional cell culture model that effectively eliminates the technical problems associated with conventional cell culture techniques. The proposed active cover lid for multi-well microplates timely addresses this issue. In the hybrid cell culture system described here, microfluidic technology is integrated with conventional multi-well microplates to achieve perfusion cell culture. Such a continuous-perfusion cell culture model not only provides a stable and well-defined cell culture environment for precise cell-based assays^17^ but also eliminates the requirement for labour-intensive manual medium replacement, which is necessary when using the conventional static cell culture method and carries associated contamination risks. Devices based on similar conceptual ideas have been presented previously for liver tissue engineering^22^ and for hepatocyte culture^23^. To the best of our knowledge, however, the delivery of medium in these functional cover lids still relies on bench-top liquid pumping equipment. In the functional cover lid described in this study, unlike those devices, a microfluidic-based mechanism for liquid transport and control is incorporated in which pneumatic pressures are utilized to simultaneously achieve multi-channel liquid delivery and control. The design makes the overall experimental setup simple and compact.

The abovementioned feature allows the scientists to carry out perfusion cell culture models in a more efficient, high-throughput, and user-friendly manner than is possible with the existing perfusion cell culture practices. Compared with the emerging microfluidic-based cell culture models, moreover, the utilization of multi-well microplate-based perfusion cell culture formats particularly enables investigators to perform initial sample loading, periodic microscopic observations, and final biochemical assays in an efficient and, user-friendly manner through the use of various types of multi-well microplate-compatible equipment (e.g., automatic liquid handling machines, functional microscopic stages, and microplate readers). This feature will potentially accelerate the widespread application of perfusion cell culture models in cell-based assays.

### Pneumatically driven multi-channel medium delivery mechanism

To achieve high-throughput perfusion 3-D cell culture-based assays, the perfusion of cells with culture medium using multi-channel and backflow-free liquid transport is required. With the current rapid progress in microfluidic technology, a wide variety of liquid transport mechanisms have been actively proposed to perform liquid delivery; these proposals have been thoroughly reviewed previously^9^. Among them, the pneumatically driven membrane-based liquid delivery mechanism that was first demonstrated by Unger and co-workers^24^ is particularly promising. The working principle of this mechanism is based on the pulsating movements of multiple elastic membranes that are pneumatically actuated and modulated by their corresponding pneumatic chambers to generate a continuous peristaltic-like activation effect for driving and controlling a fluid flow. In addition to its low cost and simplicity of fabrication and operation, a key advantageous feature of this design is that it is potentially capable of providing simultaneous multi-channel liquid delivery^9^. Based on the same working principle, several studies have successfully demonstrated the integration of multiple (6^25^, 15^26^, or 30^27^ units) pneumatic micropumps in a microfluidic system for multiplex liquid transport.

For liquid transport based on the aforementioned mechanism, one pneumatic source is normally required to simultaneously actuate multiple micropumps for multi-parallel uniform liquid transport. The rationale behind this phenomenon is that a given air flow (pressure) can be more uniformly distributed into the compartments of a manifold if the fluidic resistance of each sub-channel is uniform compared with a liquid flow counterpart^21^. This is mainly due to the low viscosity of air flow, as discussed below. For a hydrodynamic pressurizing mechanism, an applied pressure drop is a function of the fluidic resistance and the flow rate:


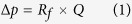


where Δ*p*, *R*_f_, and *Q* denote the pressure drop, the fluidic resistance and the flow rate, respectively. For a rectangular channel, the fluidic resistance is given by^28^


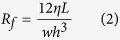


where *η*, *L*, *w*, and *h* represent the fluid viscosity and the channel length, width, and depth, respectively. From Equation (2), the viscosity nature of a fluid (*η*) determines the fluidic resistance of such fluid flow in a microchannel. The viscosity of air is approximately one fiftieth that of water. For given flow channel dimensions (e.g., the *L*, *w*, and *h*), the fluidic resistance of an air flow would be much smaller than that of a liquid flow (equation 2). Under a given pneumatic pressure, therefore, the influence of very small differences in the fluidic resistance (e.g., due to fabrication defects) on the flow rate (*Q*) in an air flow would be less significant than the influence of these differences on a liquid flow (equation 1). That is, an applied pneumatic pressure in a closed system can distribute more evenly within the whole system than a liquid flow.

**Figure 1:**
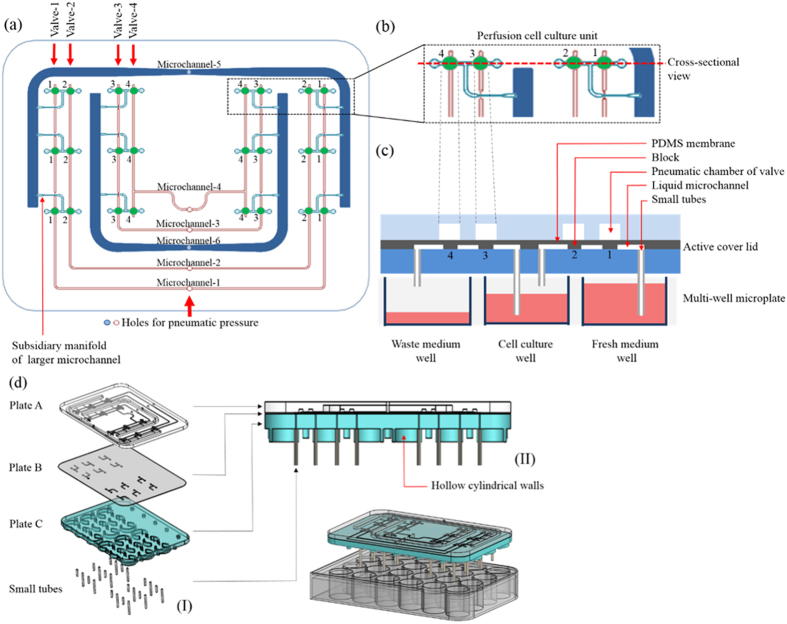
(**a**) Top-view layout of the active cover lid. (**b**) Top-view layout and (**c**) cross-sectional view of each perfusion cell culture unit. (**d**) Structure of the active cover lid: (I) assembly of the active cover lid (Plates A, B and C: microfabricated PDMS layer and 24 small tubes) and (II) cross-sectional view of the laminate structure. (These figures were drawn by Tzu-Keng Chiu).

**Figure 2:**
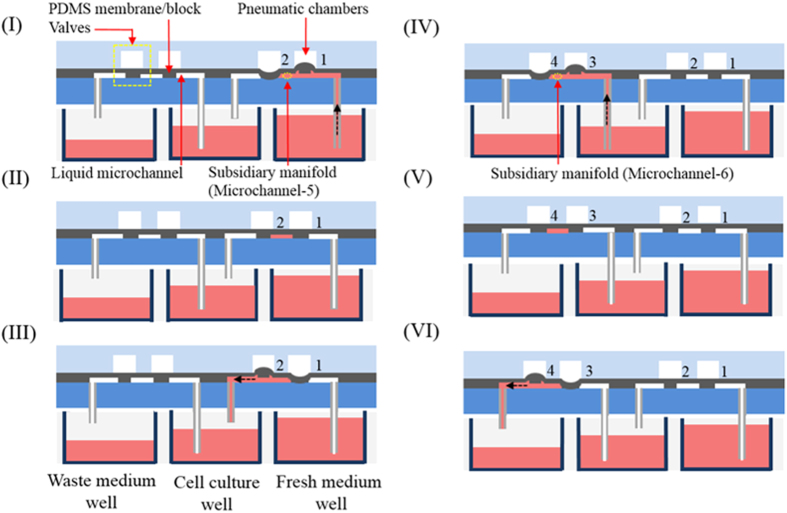
Illustration of the integrated, pneumatically driven cell culture medium delivery mechanism (the cross-sectional view of each perfusion cell culture unit). (I) Pneumatic pressure is used to open and close Valve-1 and Valve-2. Meanwhile, negative pneumatic pressure is applied to vertically suck the culture medium in the fresh medium well of the microplate to the liquid microchannel in the active cover lid. (II) The normally closed state of Valve-1 and Valve-2 after the release of the applied pneumatic pressure. (III) The use of pneumatic pressure to close and open Valve-1 and Valve-2, respectively, to mechanically squeeze the liquid medium in the microchannel so that it flows forwards to the cell culture well via the small tube. (IV)–(VI) The use of pneumatic pressure to drive the liquid medium from the cell culture well to the waste medium well [a process symmetrical to that shown in Fig. 2-(I) to (III)] (This figure was drawn by Tzu-Keng Chiu). A video clip is provided as a supplementary material, the top view.

**Figure 3:**
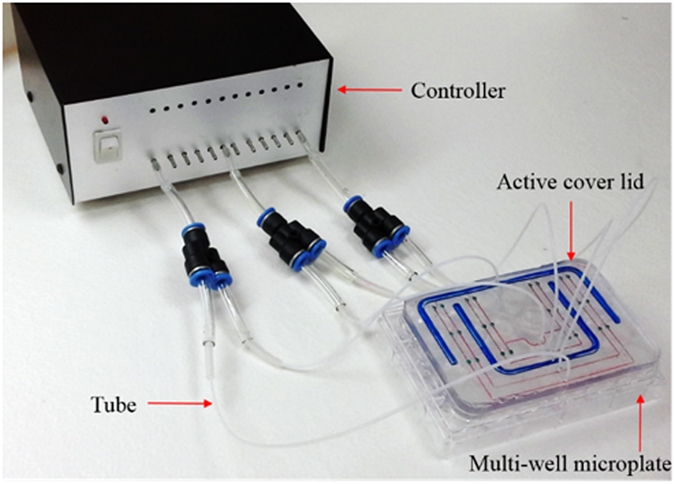
Photograph of the overall experimental setup.

**Figure 4:**
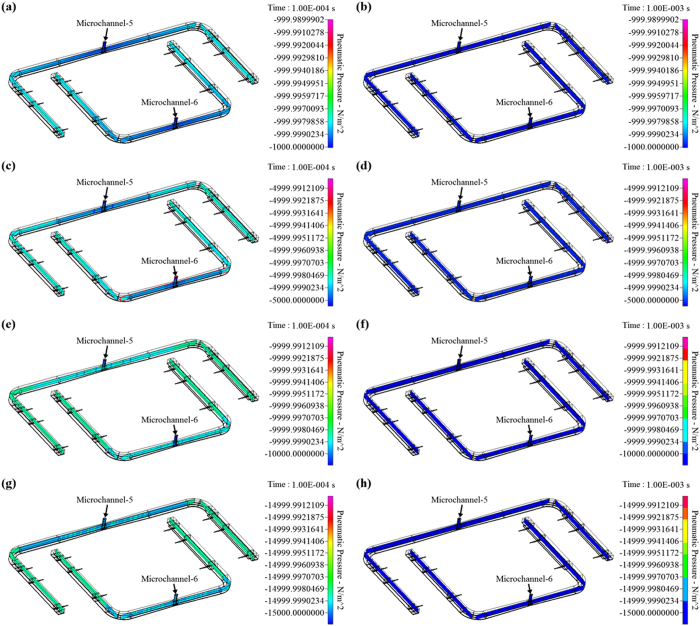
Transient state-based simulation results showing the pneumatic pressure distribution in Pneumatic microchannels-5 and -6 under different pneumatic pressure conditions [(a,b): −1 kPa; (c,d): −5 kPa; (e,f): −10 kPa; (g,h): −15 kPa] and time frame (1.0E-4 sec: the left column; 1.0E-3 sec: the right column).

Borrowing from the phenomenon described above, we designed a device in which pneumatic pressures are used to concurrently actuate and control multi-channel cell culture medium flow in the proposed active cover lid. Overall, the medium flows were activated and modulated by the coordination of pneumatically driven (1) non-mechanical and (2) mechanical liquid delivery mechanisms. For the non-mechanical liquid transport work, negative pneumatic pressure was exerted in each larger pneumatic microchannel (namely Microchannel-5 or -6: Fig. 1(a)) to vertically suck the culture medium in the well (fresh medium well or cell culture well, respectively; Fig. 1(c)) of the microplate to the liquid microchannel in the active cover lid. Each negatively pressurized larger pneumatic microchannel can concurrently drive the 6 aforementioned medium flows through 6 subsidiary manifolds (Fig. 1(a)). Meanwhile, the pneumatically driven cell culture medium delivery mechanism and overall experimental setup are shown in Figs 2 and 3, respectively. To ensure that the distribution of pneumatic pressure in the pneumatic microchannels and their subsidiary manifolds is uniform, a CFD simulation was conducted. Fig. 4 shows the transient state-based simulation results of the pneumatic flow development in Pneumatic microchannels 5 and 6 under various pneumatic pressure conditions (−1 ~ −15 kPa). It can be observed that some regions with tiny pressure differences were present in the 2 pneumatic microchannels at 1.0E-4 sec (Fig. 4(a,c,e)), indicating that the pneumatic flow continued to develop in the microchannels under all of the pneumatic conditions explored. This implies that unsteady and non-uniform pneumatic pressure distribution occurred in the microchannels at this time point. Conversely, at 1.0E-3 sec (Fig. 4; the right column), the applied pneumatic pressures (−1 ~ −15 kPa) were distributed homogenously within the 2 pneumatic microchannels, as can be clearly seen in Fig. 4(b,d,f). Within the experimental designs investigated, this indicates that the given pneumatic pressures were capable of promptly (1.0E-3 sec) and uniformly distributing within the microchannels and within their corresponding subsidiary manifolds. As a whole, the simulation results are consistent with the aforementioned speculation that an applied pneumatic pressure can be more evenly distributed within a closed system.

**Figure 5:**
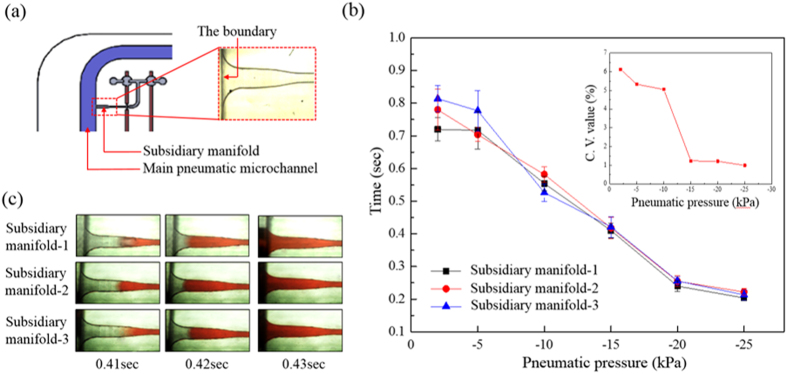
(**a**) Schematic indication of the defined boundary of the subsidiary manifold and the main pneumatic microchannel (right image: close-up micrograph). (**b**) Measured times required for the culture medium to reach the defined boundary of the 3 subsidiary manifolds under different pneumatic pressure conditions (−2 to −25 kPa). Inset: Coefficient of variation (C.V.) of the aforementioned times required for the 3 subsidiary manifolds tested under different pneumatic pressure conditions (−2 to −25 kPa). (**c**) Sequential microscopic images of liquid flows in the 3 subsidiary manifolds under different time frames (0.41, 0.42, and 0.43 sec) (Given pneumatic pressure: −15 kPa).

In addition to the above simulations, additional experiments were conducted to determine the pneumatic pressure required to achieve uniformity of the 6 flows of medium. In this work, direct microscopic observation was carried out to evaluate the time required for the culture medium to be pneumatically sucked from a fresh medium well to the boundary of the subsidiary manifold and the main pneumatic microchannel, as indicated in Fig. 5(a). Because 3 subsidiary manifolds were symmetrically designed on the left and right sides of each pneumatic microchannel, 3 subsidiary manifolds were tested in this evaluation. Figure 5(b) shows the times required for the culture medium to reach the aforementioned boundary (Fig. 5(a)) of the 3 subsidiary manifolds under different pneumatic pressure conditions (−2 to −25 kPa). It was not unexpected that the time decreased with increase in the magnitude of pneumatic pressure. To further evaluate the uniformity of flow rates of the medium vertically driven from a well to the liquid microchannel in the cover lid at these pneumatic pressures, the coefficient of variation (C.V.) of the time required for medium to reach the boundary of the 3 subsidiary manifolds tested was calculated based on Fig. 5(b). The results (inset to Fig. 5(b)) show that the C.V. value decreased with increased magnitude of pneumatic pressure. When the magnitude of applied negative pneumatic pressure was greater than 15 kPa, the C.V. value reached a low level of 0.99–1.03%, indicating that highly uniform flow rates were achieved at the pneumatic conditions. Based on the evaluations, −15 kPa of air pressure was applied in the 2 larger pneumatic microchannels (namely Microchannel-5 and -6: Fig. 1(a)) to non-mechanically suck the culture medium in the well (fresh medium well or cell culture well, respectively; Fig. 1(c)) of the microplate to the liquid microchannel in the active cover lid. After the magnitude of negative air pressure was determined, the appropriate imposition time of pneumatic pressure was also evaluated using a microscope with an associated high-speed CCD. Fig. 5(c) shows sequential images of liquid flows in the 3 subsidiary manifolds under a pneumatic pressure of −15 kPa. Overall, the microscopic images show that the liquid flow rates in the 3 subsidiary manifolds were uniform at the given air pressure of −15 kPa. Based on the observations, an imposition time of 0.41 sec was used in this study to prevent suction of liquid medium into the larger pneumatic microchannel, as shown in Fig. 5(c).

**Figure 6:**
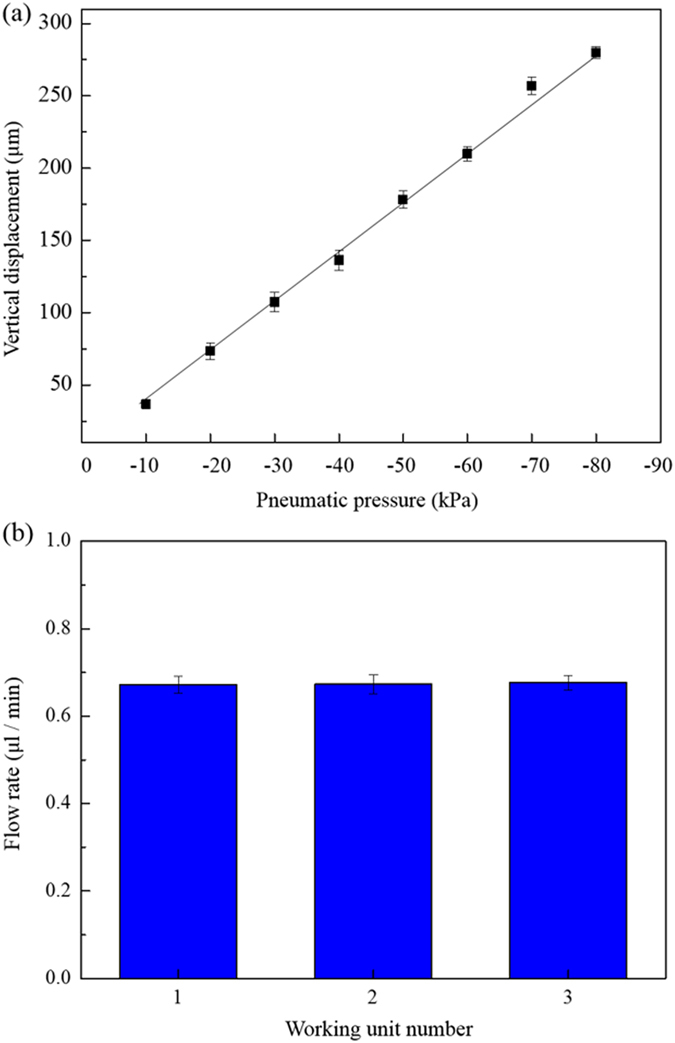
(**a**) Quantitative relationship between the negative pneumatic pressure (−10 to −80 kPa) exerted and the vertical displacement of the PDMS block from the bottom of the liquid microchannel. (**b**) The average flow rates of 3 perfusion cell culture units tested under the same operating conditions.

With respect to the mechanical action of the device, the normally closed valves were actuated by negative and positive pressure to open the liquid microchannels in the active cover lid and to mechanically squeeze the liquid in the liquid microchannels forward, respectively. To determine adequate pneumatic conditions to accomplish this, experimental evaluations were conducted. The results (Fig. 6(a)) show that there is a quantitative relationship between the negative pneumatic pressure (−10 to −80 kPa) exerted and the vertical displacement of the PDMS block from the bottom of the liquid microchannel. Within the experimental conditions explored, overall, the results were consistent with previous findings that showed the proportional relationship between the magnitude of pneumatic pressure exerted and the deformation of the PDMS membrane^29^. In this study, a negative pneumatic pressure of −50 kPa (vertical displacement of the PDMS block: 178 μm; Fig. 6(a)) was used; this pressure can effectively unblock the passageway of the liquid microchannel (H: 150 μm). For the exertion of positive pneumatic pressure to drive the liquid flow, an air pressure of 20 kPa was adopted based on our microscopic observations (images not shown) that showed that the actuation of liquid flow was compromised when the air pressure was lower than that value. After the aforementioned critical operating parameters were determined, the working frequency of all the pneumatically driven actions was set at 1 Hz, and the uniformity of the medium pumping rates of 3 working units was experimentally evaluated. Figure 6(b) shows the average flow rates of 3 working units under the given operating conditions. It can be seen that the pumping rates of the 3 working units showed high uniformity (C.V. value: 1.1%); there was no significant difference (*p* > 0.05; ANOVA) among them. Overall, the CFD simulations and experimental evaluations demonstrated that the design of the proposed active cover lid and the operating conditions used can achieve multi-parallel uniform liquid transport. In terms of practical application, moreover, it is recommended to use a surfactant solution to treat the liquid microchannel (e.g. contact with Pluronic^®^ F68 solution for up to 3 hrs), facilitating the PDMS surface to wet with aqueous solution.

### Effect of cell culture models on the viability, proliferation and osteogenic differentiation of MSCs

**Figure 7:**
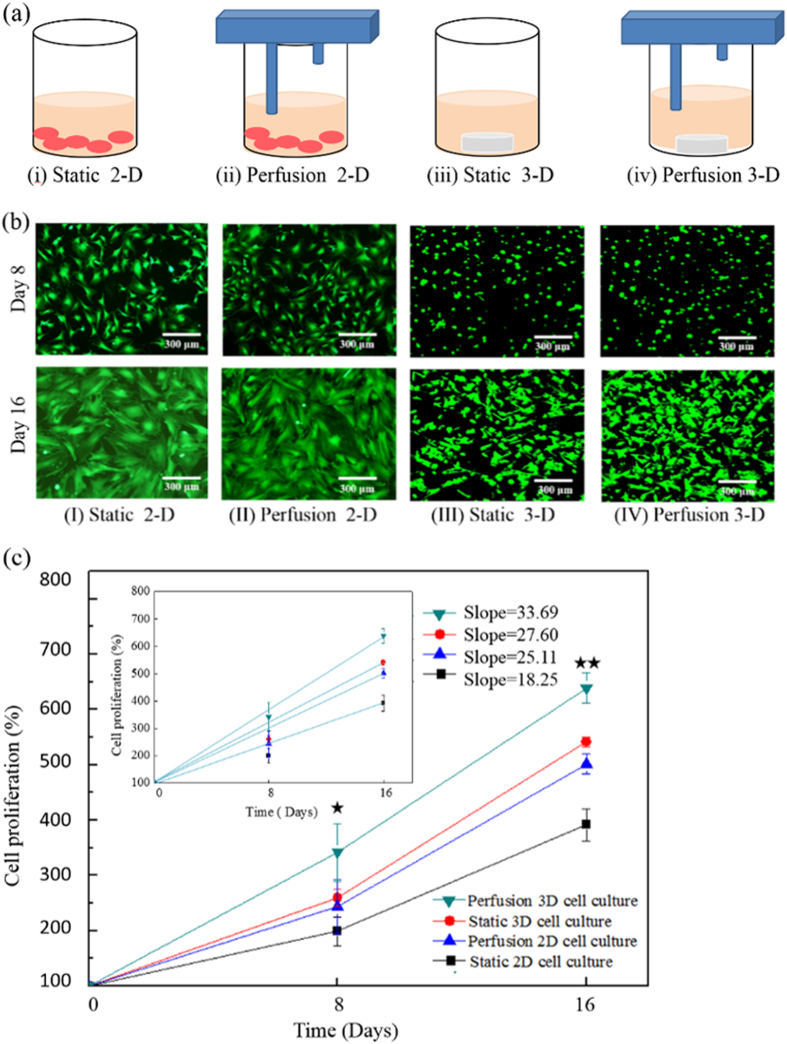
(**a**) Schematic representation of the cell culture models investigated in this study (This figure was drawn by Tzu-Keng Chiu). (**b**) Fluorescent microscopic photographs of MSCs cultured in the 4 culture models studied [(I) Static 2-D cell culture; (II) Perfusion 2-D cell culture; (III) Static 3-D cell culture; and (IV) Perfusion 3-D cell culture] on days 8 (upper row) and 16 (lower row); the green and red images represent live and dead cells, respectively. (**c**) Proliferation curves of MSCs cultured in the cell culture models tested; significant differences are indicated by stars (^**^p<0.01, ^*^p<0.05, ANOVA). Inset: Regression lines of the proliferation curves; the slope of each regression lines represents the cellular proliferation rate (% day^−1^).

Borrowing from the concept of tissue engineering, there is growing belief that perfusion 3-D cell culture models are capable of providing well-controlled, and biomimetic culture conditions for precise and physiologically relevant cell-based assays^18^. The utilization of the proposed active cover lid particularly facilitates the performance of such tasks and subsequent observations and biochemical assays in a high-throughput, user-friendly, and cost-effective manner. When a novel cell culture technique is adopted, however, the interpretation of the resulting data is demanding in terms of reconciling differences with data obtained through conventional cell culture techniques. Because living cells are quite sensitive to the extracellular biophysical^30^ or biochemical^31^ environment, it might not be appropriate to simply extrapolate data obtained from one cell culture model to data obtained using another model. To bridge the gap between data obtained using novel and conventional cell culture methods, basic studies are required to avoid any misinterpretation of data. To address these issues, several fundamental studies have been conducted to investigate the extent to which cellular physiology is influenced by various cell culture models^21^,^32^. To the best of our knowledge, however, the impact of cell culture format on the viability, proliferation and osteogenic differentiation of MSCs has not yet been fully explored. As a demonstration case, we investigated the effect of 4 different cell culture models: (i) a static 2-D cell culture; (ii) a perfusion 2-D cell culture; (iii) a static 3-D cell culture; and (iv) a perfusion 3-D cell culture, all of which are schematically illustrated in Fig. 7(a), on the viability, proliferation, and osteogenic differentiation of MSCs.

In our study, a 16-day culture duration was employed to ensure adequate time for the MSCs to undergo osteogenic differentiation^33^. In addition, the MSCs were cultured in an induction medium^33^ that was proven to be effective in inducing osteogenic differentiation of MSCs in our preliminary tests. Fig. 7(b) shows fluorescent microscopic photographs of MSCs cultured in the 4 culture models at days 8 and 16; in these photomicrographs, the green and red images represent live and dead cells, respectively. Under the experimental conditions tested, the cells retained overall viabilities as great as 96±2%. In the 2-D monolayer cell culture models (Fig. 7(b)–(I) and (II)), the cells were partially attached to the bottom surfaces of the wells on day 8, whereas on day 16 they were fully attached and spread on the surfaces. Moreover, the maintenance of cells in perfusion culture did not seem to cause significant morphological changes in the cells (Fig. 7(b)–(I) and (II)), which suggests that the fluidic shear stress on cells caused by the medium perfusion is mild in this study. In the 3-D culture models (Fig. 7(b)–(III) and (IV)), the cells exhibited spherical shapes on day 8 due to their physical encapsulation in the 3-D matrix. At day 16, however, it was observed that the cells tended to partially attach and spread within the 3-D matrix. Fig. 7(c) shows the proliferation (%) of the MSCs (the percentage of the total DNA content of cells at a particular time point relative to its initial DNA content) as a function of time. At each time point investigated (days 8 and 16), the cell culture model used had a significant effect on the proliferation of MSCs (day 8: *p* < 0.05; day 16: *p* < 0.01; ANOVA). On day 16, the proliferation of the MSCs in the perfusion 3-D cell cultures was significantly higher than the proliferation of MSCs grown under the other culture conditions (*p* < 0.01). Within the experimental conditions explored, moreover, the proliferation of cells in the perfusion 2-D and static 3-D cell culture models showed no significant difference (*p* > 0.05). Furthermore, the proliferation of cells in the perfusion 2-D and static 3-D cell culture models was significantly higher than that in the static 2-D cell culture (*p*<0.01). The slopes of the regression lines in the cell proliferation curves [inset in Fig. 7(c)] indicate the cell proliferation rates (perfusion 3-D, static 3-D, perfusion 2-D, and static 2-D cultures: 33.69, 27.60, 25.11, and 18.25%/day, respectively). Within the experimental conditions studied, the use of perfusion or 3-D cell culture formats might play a positive role in the proliferation of MSCs. The reasons for this are complex and may be related to the specific biochemical or biophysical microenvironments created under these culture conditions, which may particularly stimulate the proliferation of MSCs. The finding of a higher proliferation rate in the 3-D cell culture models tested is inconsistent with the findings of a previous report that showed that cells cultured in a 2-D monolayer culture proliferated faster than cells in a 3-D environment^34^. One possible cause of the difference in our results is that the MSCs tended to attach within the 3-D biomaterial matrix, thereby creating a quasi-2-D monolayer cell culture, as shown in Fig. 7(b)–(III) and (IV). In addition, a 3-D environment could provide more space for the cells to proliferate, whereas cell contact inhibition could occur in the 2-D cell culture models; 2-D culture models might also to some extent spatially restrict cell proliferation, as illustrated in Fig. 7(b). Overall, we conclude that the proliferation rate of MSCs varies as a function of the cell culture model used.

**Figure 8:**
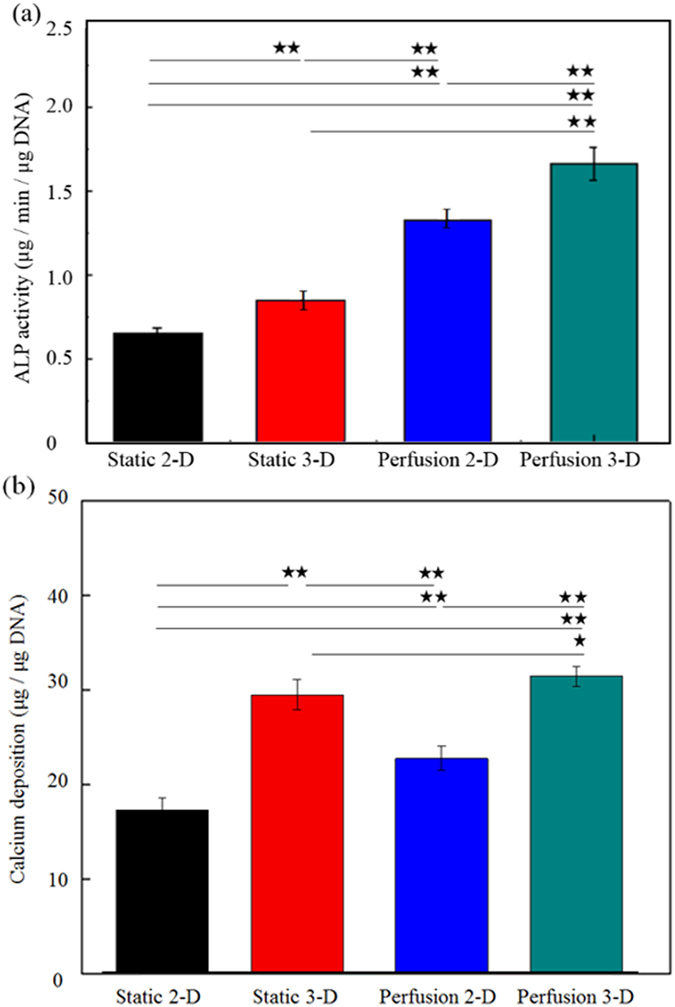
Evaluation of the osteogenic differentiation of MSCs cultured under the 4 cell culture models by measurement of (**a**) ALP activity and (**b**) calcium deposition in cells; significant differences are indicated by stars (^**^p<0.01, ^*^p<0.05, ANOVA).

To evaluate the osteogenic differentiation of MSCs cultured in the 4 cell culture models explored, two commonly used indicators, ALP activity and calcium deposition in the cells^35^, were assayed. As a whole, the results (Fig. 8(a,b)) indicate that the choice of cell culture format has a significant influence on the osteogenic differentiation of MSCs (*p* < 0.05; ANOVA). With respect to the ALP activity of the cells, the results (Fig. 8(a)) show that ALP activity differed significantly (*p* < 0.01) in all of the cell culture models; the MSCs in the perfusion 3-D cell culture model showed the highest ALP activity. A similar trend was observed in calcium deposition in MSCs (Fig. 8(b)), except that calcium deposition in MSCs in the perfusion 2-D cell culture models was significantly lower (*p* < 0.01) than calcium deposition in MSCs in the static 3-D models. Taken together, it can be concluded from the above findings (Fig. 8(a,b)) that culture of cells in the perfusion or 3-D cell culture models can upregulate the osteogenic differentiation of MSCs. Further investigation will be required to determine whether the choice of 3-D scaffolding biomaterials plays a role in the observed differences.

## Methods

### Design and principle

The proposed pneumatically driven active cover lid integrates the basic function of the top lid of a 24-well microplate and the advanced function of culture medium delivery between the wells of the microplate. A top-view layout of the cover lid is shown schematically in Fig. 1(a). The active cover lid (L: 115.3 mm; W: 75.0 mm) consists of 2 larger pneumatic microchannels (marked in blue), 4 smaller pneumatic microchannels (marked in red), and 24 pneumatically driven membrane-based, normally closed valves (marked in green). The 2 larger pneumatic microchannels (Microchannel-5: L: 173.7 mm, H: 2.0 mm; Microchannel-6: L: 147.4 mm, H: 2.0 mm) (Fig. 1(a)) with 2 corresponding through-holes (D: 1.5 mm) for applying negative pneumatic pressures were designed for perpendicularly sucking the cell culture medium in the wells of microplate to the liquid microchannels in the active cover lid through small tubes. Each larger pneumatic microchannel (Microchannel-5 or -6) was designed to simultaneously drive the 6 aforementioned medium flows through 6 subsidiary manifolds (marked in light blue) (Fig. 1(a)). In addition, the 4 smaller pneumatic microchannels (Microchannel-1: L: 199.7 mm, W: 0.50 mm, H: 0.2 mm; Microchannel-2: L: 177.6 mm, W: 0.50 mm, H: 0.20 mm; Microchannel-3: L: 125.0 mm, W: 0.5 mm, H: 0.2 mm; and Microchannel-4: L: 112.9 mm, W: 0.5 mm, H: 0.2 mm) (Fig. 1(a)) with their independent through-holes (D: 1.5 mm) were designed to apply pneumatic pressure to drive and control the action of the 24 normally closed valves (Pneumatic chamber; D: 2.0 mm; H: 1.0 mm) to manipulate the medium flows in the liquid microchannels in the active cover lid. As a whole, the active cover lid encompasses 6 working units, of which each unit (Fig. 1(b)) can be used to create a set of perfusion cell cultures. In each perfusion cell culture unit (Fig. 1(b)), the flow of medium can be pneumatically driven and controlled so that medium flows from a well containing fresh medium to a cell culture well; finally, the culture medium in the cell culture well can be delivered to a waste medium well. Figure 1(c) shows a cross-sectional view of the perfusion cell culture unit. The structure of the active cover lid is illustrated in Fig. 1(d)–(I).

To prepare the cover lid, 3 layers of microfabricated PDMS (polydimethylsiloxane) plates (A, B, and C) were first permanently bonded, followed by assembly of 24 small tubes (ID: 0.5 mm) to form a laminate structure with a cross-sectional view as shown in Fig. 1(d)–(II). The pneumatic microchannels and chambers are located in Plate A. Plate B is a thin PDMS membrane in which the microchannels (H: 0.15 mm, W: 0.5 mm) for medium flow and the moving parts [namely, the PDMS membrane (thickness: 0.3 mm) and block (L: 1.0 mm, W: 0.5 mm, H: 0.15 mm) as indicated in Fig. 1(c)] of the normally closed valves for controlling the liquid flow were fabricated. Plate C not only acts as the fourth wall of the liquid microchannels in the active cover lid but also functions as the base substrate to accommodate the 24 small tubes. In addition, 18 hollow cylindrical walls were also fabricated on the bottom side of Plate C for convenience of positioning (Fig. 1(d)–(II)).

The key technical feature of the proposed active cover lid is its ability to perform simultaneous multi-channel medium delivery for 6-unit perfusion cell cultures. The working principle is based on the combination of pneumatically driven (1) non-mechanical and (2) mechanical medium delivery mechanisms. Figure 2 illustrates the detailed culture medium pumping mechanism of each working unit. Briefly, a negative pneumatic pressure is applied in pneumatic microchannel-1 (Fig. 1(a)), through which the pneumatic chambers of Valve-1 are negatively pressurized (Figs 1(a) and 2). This forces the elastic PDMS membrane/block of Valve-1 to deform inward and thus opens the normally closed valves (Fig. 2–(I)). Meanwhile, positive pneumatic pressure is applied in Pneumatic microchannel-2 (Fig. 1(a)) by which, conversely, the PDMS membrane/block of Valve-2 deforms outward and closes the passageway of the liquid channel (Fig. 2–(I)). Shortly following the action of Valve-1 and Valve-2, negative pneumatic pressure is exerted in Pneumatic microchannel-5 (Fig. 1(a)) to suck the liquid medium in the fresh medium well to prime the liquid microchannel in the active cover lid via the subsidiary manifolds that extend perpendicularly through the liquid microchannel (Fig. 2–(I)). The pressure in Pneumatic microchannel-1 and -2 (and accordingly in Valve-1 and Valve-2) is then released, and the deformed PDMS membranes/blocks in Valves-1 and -2 return to their normal closed states (Fig. 2–(II)). This action is soon followed by the next movement, in which Valves-1 and -2 are positively and negatively pressurized to close and open the valves, respectively (Fig. 2–(III)). This movement not only mechanically squeezes the liquid medium in the microchannel, causing it to flow forward to the cell culture well via the small tube, but also prevents backflow of the culture medium to the original fresh medium well, as illustrated in Fig. 2–(III). Again, the pressures applied to Valves-1 and -2 are released, and the 2 valves return to their normal closed state (Fig. 2 (IV)). Meanwhile, Valves-3 and -4 begin their action through the pressurization of Pneumatic microchannels-3 and -4, causing the medium to flow from the cell culture well to the waste medium well (Figs 1(a) and 2 (IV)). In the process, the suction of liquid medium from the cell culture well to the liquid microchannel in the active cover lid is driven via the negative pressure applied to Pneumatic microchannel-6 (Figs 1(a) and 2(IV)). The entire process (Fig. 2–(IV) to (VI)) is symmetrically the same as the aforementioned process (Fig. 2-(I) to (III)) that delivers liquid medium from the fresh medium well to the cell culture well.

### Microfabrication and experimental setup

In this study, PDMS was used as material to construct the active cover lid because it is gas-permeable, non-toxic, and biocompatible. Due to the gas-permeable feature of PDMS, the gas condition (e.g. CO_2_ or O_2_) in the wells of microplate can be manipulated through the surrounding gas condition in the cell incubator. The overall fabrication process was based on a computer numerical controlled (CNC) machining process, PDMS replica moulding, and a plasma oxidation–aided bonding process. These are fundamental microfabrication techniques which are suitable for the use in low-quantity production (e.g. for academic researches). Briefly, PDMS Plates A, B, and C (Fig. 1(d)–(I)) were fabricated using a combination of CNC machining and PDMS replica moulding. First, three positive polymethylmethacrylate (PMMA) moulds for PDMS Plates A, B, and C were fabricated using a CNC miller (EGX-400, Roland, Japan) and a 1.0-mm drill bit (rotational speed: 23,000 rpm). In the subsequent replica moulding process, PDMS (Sylgard^®^ 184, Dow Corning, USA) was prepared by thoroughly mixing the PDMS pre-polymer with a curing agent (10:1, w/w), poured onto the fabricated PMMA moulds and then cured at 70 °C for 1 hr. For PDMS Plate C, the multiple through-holes for the insertion of the small tubes (Fig. 1(d)–(I)) were formed directly by casting the PDMS polymer with its level lower than the height of the cylindrical moulds on its PMMA mould. The cured PDMS Plates A, B, and C were then obtained through a careful de-moulding process. In the following assembly process, the 3 microfabricated PDMS plates were permanently bonded through plasma oxidation treatment followed by assembly with the 24 small tubes through physical insertion to form the active cover lid. To drive and control the pneumatically driven active cover lid, 6 holes on the pneumatic microchannels (Fig. 1(a)) were connected with 6 air tubes from a custom-made controller in which a vacuum pump, a compressor, digital pressure regulators, 6 electromagnetic valves (EMVs; S070 M-5BG-32, SMC, Taiwan), and a programmable control circuit were integrated. A photograph of the overall experimental setup is shown in Fig. 3.

### Computational fluid dynamics (CFD)-based simulation to evaluate the pneumatic pressure distribution in the pneumatic microchannels

As described in Fig. 2, negative pneumatic pressure was used to vertically draw the culture medium in the wells of the microplate into the liquid microchannel in the active cover lid. In the design, a pneumatic pressure was applied in the pneumatic microchannel (Microchannel-5 or Microchannel-6; Fig. 1(a)) to simultaneously drive the 6 aforementioned medium flows through 6 subsidiary manifolds (Fig. 1(a)). To ensure uniform distribution of pneumatic pressure in the pneumatic microchannels (Microchannel-5 and Microchannel-6), computational fluid dynamics (CFD)-based simulation was employed as a design aid. Briefly, the CFD technique was used to solve the full 3-D geometry by Navier-Stokes equations for the air flow in pneumatic Microchannels-5 and -6. Numerically speaking, the conservation equations were solved by the finite volume method (FVM) with SIMPLEC (Semi-Implicit Method for Pressure-Linkage Equations Consistent) pressure-velocity coupling. The original method, SIMPLE, was created by Van Doormal and Raithby^36^. SIMPLEC is an extension of the SIMPLE method and is derived from the finite difference form of the momentum equation. The scheme of FVM coupling with the SIMPLEC iterative solver was based on the cell midpoints, and forward/backward Euler was used on the boundary nodes. It was implemented in the CFD-ACE+ multiphysics suite (ESI Group, Paris, France). Meanwhile, the mesh generation for the 3D geometry was conducted using CFD-GEOM (ESI Group, Paris, France), leading to a structured Cartesian mesh. The geometry of the 3D pneumatic-driven device has 1298497 nodes and 1023692 cells.

### Evaluation of operating conditions and performance of the integrated liquid delivery mechanism

In the liquid delivery mechanism (Fig. 2), the culture medium in the fresh medium well (or cell culture well) is pneumatically driven to the liquid microchannel in the active cover lid in a non-mechanical manner. As described earlier (Fig. 1(a)), negative pneumatic pressure is exerted in the larger pneumatic microchannel (Microchannel-5 or -6) to simultaneously drive the 6 aforementioned medium flows through the 6 subsidiary manifolds. To determine the pneumatic conditions under which the 6 flows of medium are uniform, the following evaluations were performed. Briefly, negative pneumatic pressures ranging from −2 to −25 kPa were applied in Pneumatic microchannel-5 (Fig. 1(a)). Direct microscopic observation was carried out to evaluate the time required for the culture medium to be pneumatically driven from a fresh medium well to the boundary of the subsidiary manifold and the main pneumatic microchannel. To clearly observe the aforementioned liquid medium flows, a high-speed, charge-coupled-device (CCD) camera (MC1311, Mikrotron, Germany) coupled to a microscope (TE300, Nikon, USA) was used. The resolution speed was set at 200 frames sec^−1^ to distinctly capture the relevant images. In addition to the magnitude of pneumatic pressure, the imposition time of pneumatic pressure is also a critical factor that could affect the function of the proposed liquid delivery mechanism in the active cover lid. To determine a suitable imposition time of negative pneumatic pressure, the liquid flow in a subsidiary manifold was also observed microscopically using the aforementioned method.

In the active cover lid, negative pressure was applied in the pneumatic chamber of the pneumatically driven normally closed valve to open the liquid passageway, whereas the pneumatic chamber was positively pressurized to cause the deformation of the PDMS membrane/block and thus mechanically cause the liquid in the microchannel to flow forward. To determine the appropriate pneumatic conditions for manipulation of the valves, the following experiment was conducted. First, the quantitative link between the applied negative pneumatic pressure and the vertical displacement of the PDMS block from the bottom of the microchannel was established. The vertical displacement of the PDMS block was quantified using a microscopic focusing method^26^. Briefly, the bottoms of the PDMS block and liquid microchannel were labelled with different colours. The difference in the microscopic focal length needed to clearly image the two different colours was quantified using a microscope (TE300, Nikon, USA). A known microchannel depth quantified by a profiler (Dektak 6 M Surface Profiler, Veeco, USA) was used as the reference. To determine the positive pressure needed to mechanically drive a liquid flow, microscopic observation was carried out to directly evaluate the performance of liquid transport under various positive pressure conditions. After the aforementioned operating conditions were determined, the uniformity of the medium pumping rate of each working unit was experimentally evaluated. In the evaluation, the volumetric medium pumping rates were quantified by measuring the weight of liquid output over a period of 24 hr using an electronic balance (readability: 0.1 mg, repeatability: 0.1 mg; AB54-S, Mettler Toledo, Taiwan)^26^. The measured weight of liquid output was then converted to volume of liquid, assuming a constant density of water (1 g cm^−3^). Three individual experimental cases were performed to obtain flow rate data for further statistical analysis.

### Effect of cell culture models on the viability, proliferation and osteogenic differentiation of mesenchymal stem cells (MSCs)

#### Cell culture

To investigate the extent to which cellular physiology is influenced by the adopted cell culture models, we performed the following experiments. As a demonstration case, cell viability and proliferation, as well as osteogenic differentiation, of MSCs under different cell culture models [(i) a static 2-D cell culture; (ii) a perfusion 2-D cell culture; (iii) a static 3-D cell culture; and (iv) a perfusion 3-D cell culture] were explored. Briefly, MSCs were isolated from the bone marrow of 6–8 week old rats weighing 110–120 g. The isolation process was based on a commonly used protocol that has been described previously^37^. The isolated MSCs were cultured in Petri dishes to obtain adequate numbers of cells for subsequent experiments. Ten millilitres of Dulbecco’s Modified Eagle’s Medium (DMEM) (1000 mg L^–1^ glucose, 25 mM HEPES) (unless otherwise stated, all chemicals were purchased from Sigma, Taiwan) supplemented with 10% (v/v) foetal bovine serum (FBS) (Invitrogen, Taiwan) and 1% penicillin was supplied on a 3-day basis. Only cells at the 4^th^ passage were used^38^. After expansion of the MSC cultures, the cells were harvested and cultured in the 4 aforementioned cell culture models. For the static 2-D monolayer cell culture, 1*10^5^ cells were seeded per well in 24-well microplates, and the plats were kept in a standard cell incubator for 16 days. After the first day of culture, the culture medium was changed to an osteogenic induction medium (DMEM: 1,000 mg L^–1^ glucose, 25 mM HEPES, 10% (v/v) FBS, 1% penicillin, 0.1 μM dexamethasone, 50 μM ascorbate-2-phosphate, and 10 mM β-glycerol phosphate). Three millilitres of the induction medium was supplied on a 3-day basis. For static 3-D cell culture, the MSCs were cultured in a 3-D collagen/gelatin scaffold for 16 days. Briefly, cell suspensions (1*10^5^ cells in 40 μl culture medium) were thoroughly mixed with 4 μl of 0.1% collagen suspension (Type-I collagen) followed by injection into a commercially available cylindrical 3-D gelatin hydrogel scaffold (D: 9.0 mm, H: 1.5 mm; Go-Matrix^®^, BBBMD Ltd., Taiwan) using a pipette. As was the case for the static 2-D culture models, the culture medium was changed to induction medium after one day of culture, and 3 ml of culture medium was supplied every 3 days. For the perfusion 2-D and 3-D cell culture models, preparation of the cells was performed as described above. In the 2 perfusion cell culture models, the proposed active cover lid was used to achieve continuous medium perfusion at a flow rate of 41.5 μl hr^−1^; this provided a medium supply rate approximately equivalent to that provided in the 2 static cell culture models.

#### Bioassays

The viabilities of the MSCs in the 4 different cell culture models were measured on days 8 and 16 using a fluorescent dye kit (LIVE/DEAD^®^ Viability/Cytotoxicity Kit L-3224, Molecular Probes)^21^,^26^ and confocal microscopy (LSM 510 META, Zeiss, Germany). In addition, the proliferation of MSCs cultured in the 4 different cell culture models was observed by quantifying the DNA content of the cells on days 8 and 16. The DNA was assayed using Hoechst 33258 dye and fluorometric detection^39^. Briefly, 40 μl of papain-digested sample and 200 μl of Hoechst 33258 dye (0.2 μg/ml) in TN buffer (50 mM Tris, 150 mM NaCl, pH 7.5) were mixed in a well of a 96-well microplate. The fluorescence of the mixture was then read in a fluorescence microplate reader (Infinite^®^ M1000 PRO, TECAN, Switzerland) at 360 nm excitation and 460 nm emission with a 420 nm cut-off filter. Zero to 10 ng of double-stranded calf thymus DNA was used as standard. To evaluate the osteogenic differentiation of MSCs in the 4 different cell culture models, intracellular alkaline phosphatase (ALP) activity and calcium deposition in the cells were measured after 16 days of culture. For the measurement of ALP activity^40^, the cells were harvested and centrifuged at 12,000 rpm for 10 min, and the cell pellet was assayed for ALP activity using *p*-nitrophenyl-phosphate (*p*NPP) as substrate. The cell pellets were added to single wells of a 96-well microplate, each of which contained 250 μl of ALP solution (56 mM 2-amino 2-methyl-1, 3-propanediol and 1 mM MgCl_2_ containing 10 ml of *p*NPP). The mixture was incubated at 37 °C for 30 min, followed by the addition of 250 μl of 1 N NaOH to stop the reaction. The hydrolysis of *p*NPP to *p*-nitrophenol (*p*NP) was measured spectrophotometrically at a wavelength of 405 nm. To assay calcium deposition in cells^41^, 2-D or 3-D cell culture samples were washed 3 times in distilled water and then incubated in 500 μl of 0.6 N hydrochloric acid at 4 °C for 24 hr to extract calcium. The total calcium content of each sample was spectrophotometrically measured using the o-cresophalein-complexone method according to the manufacturer’s instructions (Synergy^™^HT, Bio Tex, USA).

#### Statistical analysis

Data from at least three separate experiments were analysed and were presented as mean ± standard deviation. For a given experiment, each condition was tested in triplicate. One-way ANOVA analysis with a statistical significance level of 0.05 was used to examine the uniformity of the multi-channel flow rates, the effect of cell culture model on cell proliferation, ALP activity, and calcium deposition in cells. The Tukey Honestly Significant Difference (HSD) *post hoc* test was used to compare the differences between two conditions investigated when the null hypothesis of ANOVA analysis was rejected.

## Conclusions

We have developed a pneumatically driven active cover lid for use in multi-well microplate-based perfusion 3-D cell culture. Before the emerging microfluidic-based cell culture models can be widely employed in cell-based assays, there is an urgent need for a transitional cell culture technique that can effectively overcome the technical problems associated with the conventional static and perfusion cell culture models. The proposed hybrid cell culture system consists of an active cover lid for multi-channel culture medium perfusions, and a commercially available 24-well microplate for accommodating 3-D cell culture samples. The hybrid cell culture system particularly allows scientists to perform perfusion cell culture-based assays and subsequent analyses in an efficient, cost-effective, and user-friendly manner without the need for costly and bulky liquid pumping equipment (e.g., syringe pumps) and with the aid of commonly available multi-well microplate-compatible instruments (e.g., a liquid handling machine, a microplate reader or a functional microscope stage). Using computational simulations, it was confirmed that the pneumatic pressures exerted in the hybrid culture system were homogeneously distributed within the pneumatic microchannels over a very short time frame of 1.0E-3 sec, demonstrating the potential for the use of pneumatic pressure to simultaneously and uniformly drive 6 flows of culture medium. This was further experimentally verified, demonstrating that the flows of culture medium can be pneumatically driven in a flow-rate uniform manner [coefficient of variation (CV): 1.1%]. As a demonstration case, moreover, we successfully used the proposed system to investigate the influence of 4 cell culture models on the viability, proliferation, and osteogenic differentiation of MSCs. We found that the physiological nature of MSCs varies depending on the cell culture model used and that the use of the perfusion or 3-D cell culture format significantly upregulated the proliferation and osteogenic differentiation of MSCs. This suggests that the choice of cell culture format might lead to different evaluation outcomes. When a novel cell culture methodology is adopted, therefore, more fundamental research is required to bridge the gap between conventional and novel protocols.

## Additional Information

**How to cite this article**: Huang, S.-B. *et al.* Development of a pneumatically driven active cover lid for multi-well microplates for use in perfusion three-dimensional cell culture. *Sci. Rep.* **5**, 18352; doi: 10.1038/srep18352 (2015).
